# An audit and feedback intervention study increased adherence to antibiotic prescribing guidelines at a Norwegian hospital

**DOI:** 10.1186/s12879-016-1426-1

**Published:** 2016-02-27

**Authors:** June Utnes Høgli, Beate Hennie Garcia, Frode Skjold, Vegard Skogen, Lars Småbrekke

**Affiliations:** Department of Pharmacy, Faculty of Health Sciences, UiT – The Arctic University of Norway, N - 9037 Tromsø, Norway; Department of Infectious Diseases, Division of Internal Medicine, University Hospital of North Norway, N - 9038 Tromsø, Norway; Department of Clinical Medicine, Faculty of Health Sciences, UiT – The Arctic University of Norway, N - 9037 Tromsø, Norway

**Keywords:** Community-acquired pneumonia, Acute exacerbation of chronic pulmonary disease, Intervention, Antibiotic, Audit and feedback, Norway

## Abstract

**Background:**

Appropriate antibiotic prescribing is associated with favourable levels of antimicrobial resistance (AMR) and clinical outcomes. Most intervention studies on antibiotic prescribing originate from settings with high level of AMR. In a Norwegian hospital setting with low level of AMR, the literature on interventions for promoting guideline-recommended antibiotic prescribing in hospital is scarce and requested. Preliminary studies have shown improvement potentials regarding antibiotic prescribing according to guidelines. We aimed to promote appropriate antibiotic prescribing in patients with community-acquired pneumonia (CAP) and acute exacerbations of chronic obstructive pulmonary disease (AECOPD) at a respiratory medicine department in a Norwegian University hospital. Our specific objectives were to increase prescribing of appropriate empirical antibiotics, reduce high-dose benzylpenicillin and reduce total treatment duration.

**Methods:**

We performed an audit and feedback intervention study, combined with distribution of a recently published pocket version of the national clinical practice guideline. We included patients discharged with CAP or AECOPD and prescribed antibiotics during hospital stay, and excluded those presenting with aspiration, nosocomial infection and co-infections. The pre- and post-intervention period was 9 and 6 months, respectively. Feedback was provided orally to the department physicians at an internal-educational meeting. To explore the effect of the intervention on appropriate empirical antibiotics and mean total treatment duration we applied before-after analysis (Student’s *t*-test) and interrupted time series (ITS). We used Pearson’s *χ*2 to compare dose changes.

**Results:**

In the pre-and post-intervention period we included 253 and 155 patients, respectively. Following the intervention, overall mean prescribing of appropriate empirical antibiotics increased from 61.7 to 83.8 % (*P* < 0.001), overall mean total treatment duration decreased from 11.2 to 10.4 days (*P* = 0.015), and prescribing of high-dose benzylpenicillin decreased from 48.8 to 38.6 % (*P* = 0.125). With ITS we found that six months post-intervention, the effect on appropriate empirical antibiotic prescribing had increased and sustained, while the effect on treatment duration was at pre-intervention level.

**Conclusion:**

The combination of audit and feedback plus distribution of a pocket version of guideline recommendations led to a substantial increase in prescribing of appropriate empirical antibiotics, which is important due to favourable effect on AMR and clinical outcomes.

**Electronic supplementary material:**

The online version of this article (doi:10.1186/s12879-016-1426-1) contains supplementary material, which is available to authorized users.

## Background

Appropriate antibiotic prescribing against Community-Acquired Pneumonia (CAP) and Acute Exacerbation of Chronic Obstructive Pulmonary Disease (AECOPD) may reduce risk of treatment failure, hospital readmission, mortality and health care costs, increase quality of life, and delay development of antimicrobial resistance (AMR) [1, 2]. Appropriate antibiotic prescribing includes selection of guideline-recommended empirical antibiotic treatment, dose and total treatment duration [2, 3]. Empirical antibiotic selection in Norway is mainly based on level of AMR amongst *S.pneumoniae,* which is the commonest bacterial pathogen in CAP and AECOPD [4–6]*.* Other pathogens include *H.influenzae, M.pneumoniae* and respiratory viruses [4–6]. In Norway, <1 % of *S.pneumoniae* are resistant to benzylpenicillin [7]. For *H.influenzae*, the prevalence of beta-lactamase and chromosomal resistance is 15 % and 19 %, respectively [6, 7]. Consequently, recommended empirical prescribing in Norway for non-severe hospitalized CAP and AECOPD-patients is benzylpenicillin (low-dose 1.2 g x 4) or amoxicillin/ampicillin. In severe hospitalized patients, the recommended prescribing is benzylpenicillin (high-dose 3.0 g x 4) in monotherapy or in combination with gentamicin, or cefotaxime in monotherapy as second choice. A macrolide is added if atypical pathogens such as *M.pneumoniae* are suspected [6].

Inappropriate antibiotic prescribing related to empirical antibiotic selection and total treatment duration is frequently reported for both CAP and AECOPD patients [8, 9]. Interventions of persuasive or restrictive character to promote appropriate prescribing are equally effective after six months, but restrictive interventions have greater immediate impact [10]. Audit and feedback (A&F), applied alone or in combination with other interventions, is a persuasive strategy for improving professional practice [11]. In A&F you audit clinical practice within a specific time period and compare results with standards or targets. Healthcare professionals are then informed about the results, orally, in writing or both. The goal is adjustment of clinical practice to approach standards or reach targets [11]. Most intervention studies originate from countries with high level of AMR and a prescribing pattern that differ from Norway. In the Norwegian setting, with extensive prescribing of benzylpenicillin and low level of AMR, the literature on interventions for promoting appropriate hospital prescribing is requested [12]. The overall aim of this study was to promote appropriate antibiotic prescribing in CAP and AECOPD patients at a respiratory medicine department in a Norwegian hospital. Specific objectives were to increase prescribing of appropriate empirical antibiotics, reduce prescribing of high-dose benzylpenicillin and shorten total treatment duration.

## Method

**Fig. 1:**
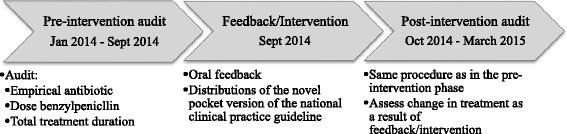
Overview of the three-phase audit and feedback intervention study

We performed a three-phase A&F intervention study at an 18-bed respiratory medicine department at the University Hospital of North Norway (UNN) Tromsø. UNN is a 500-bed hospital, and serves as local hospital for about 190 000 inhabitants. We included all patients discharged with CAP or AECOPD that were prescribed antibiotics during their hospitalization. Patients with co-infections, nosocomial pneumonia and aspiration pneumonia were excluded. See Fig. 1.

### Pre-intervention phase (January 1 – September 30, 2014)

We used the electronic hospital administrative system to identify eligible patients discharged during the nine-month pre-intervention audit. We assessed antibiotic prescribing practice by retrospectively reviewing medication charts and electronic patient records, denoting predefined data in data collection forms. For each patient we evaluated whether antibiotic prescribing was in accordance with national clinical practice guideline (CPG) recommendations, see Table 1 [6]. In the pre-intervention audit we focused on empirical antibiotic selection, dose and treatment duration. Specifically, we targeted prescribing of empirical antibiotics categorizing the prescribing as either appropriate (i.e. benzylpenicillin in monotherapy, in combination with gentamicin, or amoxicillin/ampicillin in monotherapy) or inappropriate (i.e. all other antibiotics). The proportion of appropriate empirical antibiotics maintained during first three days and during entire hospital stay were measured, but not targeted. We also targeted dose of benzylpenicillin (i.e. high-dose 3 g x 4 and low-dose 1.2 g x 4), and total treatment duration. The latter was calculated as length of inpatient prescribing plus length of prescription at discharge. We extracted data on 30-day mortality and 30-day unplanned readmission of any cause from the electronic patient journal.

**Table 1:** An abbreviated overview of the Norwegian clinical practice-guideline recommendations

Infection	Drug	Dose	Duration
AECOPD	Benzylpenicillin (intravenous) Ampicillin (intravenous) Amoxicillin (oral)	1.2 g x 4 1.0 g x 4 500 mg x 3	5 days
CAP Non-severe pneumonia	Benzylpenicillin (intravenous)	1.2 g x 4	5–7 days
CAP Severe pneumonia (CRB-65 3–4)	Benzylpenicillin (intravenous) + alternatively addition of gentamicin Cefotaxime^a^(intravenous) + alternatively addition of erythromycin (intravenous or oral)	3 g x 4 5 mg/kg x 1 1–2 g x 3 500 mg x 4	7–10 days

Valid for hospitalized patients with acute exacerbation of chronic obstructive pulmonary disease (AECOPD) and community-acquired pneumonia (CAP)

*CRB-65* Confusion, respiration, blood pressure and age > 65y. ^a^In analysis categorized as inappropriate, see discussion

### The feedback (September 2014)

The head of the respiratory medicine department summoned all the department physicians to attend the feedback session where we presented the project, the essential CPG recommendations and the results of the pre-intervention audit. In addition, we distributed the recently published pocket version of the national CPG. The physicians were encouraged to discuss audit results, especially with focus on the identified discrepancies between documented performance and the CPG recommendations. While the principal investigator (JUH) led the feedback session, an infection disease (ID)-specialist (VS) took active part in the discussion and commented on CPG recommendations and audit results.

### Post-intervention phase (October 1, 2014 – March 31, 2015)

We conducted the post-intervention audit in the same manner as the pre-intervention audit, but the audit was restricted to six months due to time constraints.

The Regional Committee for Medical and Health Research Ethics assessed the study, and concluded that the procedure was a qualitative improvement initiative and outside their remit to evaluate. The hospital’s personal data protection officer approved the study. The need for informed consent was wavered because patient data was retrospectively and anonymously extracted from patient records and consequently did not put patients at any risk, and because the physicians taking part in the study did this as part of their daily working routine. All data are aggregated data, reported anonymously, and patient confidentially is maintained.

### Statistics

We compared patient characteristics in the pre- and post intervention period using Pearson’s *χ*2-test for categorical data and Student’s *t*-test for continuous data.

The effect of the intervention on overall changes in prescribing of appropriate empirical antibiotics and total treatment duration was analysed using before-and-after analysis (Student’s *t*-test) and interrupted time series (ITS). For the latter, we evaluate both a level effect and a trend effect. Consequently, we estimate both immediate, delayed and sustained effect while taking into account the time trend [13, 14]. The regression model is given by:

$$ {\widehat{\mathrm{Y}}}_{\mathrm{t}} = {\upbeta}_0 + {\upbeta}_{1\kern0.5em }\mathrm{xtim}{\mathrm{e}}_{\mathrm{t}} + {\upbeta}_2\mathrm{xintervention} + {\upbeta}_3\mathrm{xtime}\ \mathrm{after}\ \mathrm{interventio}{\mathrm{n}}_{\mathrm{t} + }{\mathrm{e}}_{\mathrm{t}}, $$

Ŷ_t_ is the outcome, β_0_ is the intercept, β_1_ is the slope pre-intervention, β_2_ is the change in level one and six months post-intervention, β_3_ is the change in slope post-intervention compared to pre-intervention (change in trend), and e_t_ is the error estimate [13, 14]. Change in level is the difference between the last point pre-intervention and the point of interest post-intervention (e.g. first point), and the trend change can reverse or enhance a level change [15]. The analyses were controlled for autocorrelation and seasonality by applying Durbin-Watson statistics and autocorrelation function plot. For the ITS analysis, data was processed, analysed and reported in accordance to relevant guidelines [14, 16].

For comparison of dose differences pre- and post-intervention, we used the Pearson’s *χ*2 test. In all tests, a *P*-value of < 0.05 was considered statistically significant. We conducted the statistical analysis using Microsoft® Office Excel 2013 and SPSS® 22.0 for Windows.

## Results

### Patient characteristics

In the pre- and post-intervention phase we included 253 and 155 patients, respectively. A significant decrease from pre- to post-intervention was observed concerning proportion of patients with AECOPD and penicillin allergy. Following the intervention, we observed no negative effect on 30-day mortality, 30-day readmission and length of stay in hospital (Table 2). For detailed information on differences in study outcome measures for appropriate and inappropriate empirical antibiotic prescribing, see Additional file 1. For detailed information on microbiological diagnostics and findings, see Additional files 2, 3 and 4.

**Table 2:** Patient characteristics of patients included pre- and post-intervention

	Pre-intervention		Post-intervention		*P*-value
	n	(%)	n	(%)	
Study participants	253		155		
Female	126	(49.8)	75	(48.4)	NS
Age, years					NS
Mean (range)	71.4	(78)	72.1	(75)	
Median	73.0		73.0		
Infection					
AECOPD	104	(41.4)	45	(29.0)	0.014
Community-acquired pneumonia	149	(58.9)	110	(71.0)	
Nursing home residents	25	(9.9)	9	(5.8)	NS
Penicillin allergy	32	(12.6)	9	(5.8)	0.03
Risk factors for non-common bacteria or resistant bacteria					
Malignity or immunocompromised	36	(14.2)	24	(15.5)	NS
Preceding hospitalization last 30d	57	(22.5)	24	(15.5)	NS
Microbiological diagnostics					
Blood culture	172	(68.0)	125	(80.6)	0.005
Nasopharynx and/or expectorate	205	(81.0)	141	(91.0)	0.007
Pneumococcal urinary antigen test	149	(58.9)	92	(59.4)	NS
Other^a^	157	(62.1)	104	(67.1)	NS
None	16	(6.3)	5	(3.2)	NS
Aetiology					
*S.pneumoniae*	30	(11.9)	16	(10.3)	NS
*H.influenzae*	12	(4.7)	13	(8.4)	NS
Other bacteria	15	(5.9)	11	(7.1)	NS
Influenza virus A or B	24	(9.6)	18	(11.6)	NS
Other respiratory viruses	34	(13.4)	30	(19.4)	NS
None identified	159	(62.8)	89	(57.4)	NS
CRB-65 score					NS
0	25	(16.8)	16	(14.5)	
1	57	(38.3)	42	(38.2)	
2	48	(32.2)	32	(29.1)	
3	7	(4.7)	11	(10.0)	
4	2	(1.3)	1	(0.9)	
Missing data	10	(6.7)	8	(7.3)	
Clinical outcomes					
Length of stay, median (range)	5.3	(34)	5.9	(33)	NS
30 day mortality	18	(7.1)	14	(9.0)	NS
30 day readmission	55	(22.8)	24	(16.7)	NS

*AECOPD* Acute exacerbation of chronic obstructive pulmonary disease, *CRB-65* Confusion, respiration, blood pressure and age ≥ 65y, ^a^High prevalence due to urinary samples are included. For more detailed information on microbiological diagnostics, see Additional files 2, 3 and 4

### Prescribing of appropriate empirical antibiotics

During the feedback we agreed with department physicians to target increased prescribing of appropriate empirical antibiotics. Following the intervention, the overall mean prescribing of appropriate empirical antibiotics increased from 61.7 to 83.8 %, *P* < 0.001. The most prominent changes were appropriate antibiotics replacing doxycycline in AECOPD-patients and cephalosporines in CAP-patients (Table 3).

**Table 3:** Comparison of empirical antibiotic prescribing pre- and post-intervention, expressed as percentage-point difference

Antibiotic agent	Pre-intervention (%)	Post-intervention (%)	Percentage-point difference
Benzylpenicillin^a^	41.1	53.5	+12.4
Benzylpenicillin + gentamicin^a^	8.3	16.1	+7.8
Ampicillin and amoxicillin^a^	12.3	14.2	+1.9
Cephalosporines^b^	16.2	9.7	−6.5
Tetracyclines^b^	14.6	3.2	−11.0
Macrolides	2.8	-	−2.8
Others	4.7	3.2	−1.9

Proportion of patients prescribed the specific antibiotics is calculated based on number of study participants pre- and post-intervention (*n* = 253 and 155, respectively)

^a^Categorized as appropriate, and targeted for increase, ^b^For cephalosporines and doxycyclines percentage difference was −3.8 % and −21.6 % in patients with acute exacerbation of chronic obstructive pulmonary disease and −11.2 % and −2.0 % in patients with community-acquired pneumonia, respectively

**Fig. 2:**
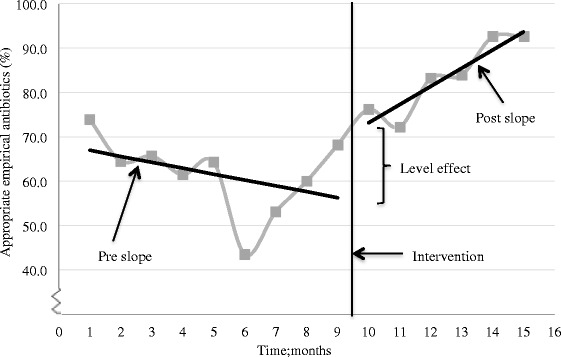
Trend and level change in monthly prescribing of appropriate empirical antibiotics, pre- and post-intervention. Appropriate antibiotics comprise benzylpenicillin in monotherapy, benzylpenicillin in combination with gentamicin, or amoxicillin/ampicillin. Pre-intervention audit started January 2014 (point no.1), the intervention was performed late September 2014 (point no. 9) and the post-intervention audit ended March 2015 (point no. 15)

Using the monthly proportion of appropriate empirical antibiotics prescribed, we analysed trend and level change with ITS. Pre-intervention, the prescribing slope of appropriate empirical antibiotics non-significantly decreased 1.3 % per month. The immediate effect of the intervention was non-significant (estimated level change; +14.1 %, *P* >0.05). However, post-intervention, the trend of appropriate empirical antibiotics significantly increased (trend change; + 4.1 % per month, *P* = 0.02), and six months post-intervention the effect of the intervention was significant (estimated level change; +45.4 %, *P* = 0.002*)*. See Fig. 2 and Table 4.

**Table 4:** Monthly prescribing of appropriate empirical antibiotics and total treatment duration, estimated with interrupted times series

	Appropriate empirical antibiotics^a^			Total treatment duration		
	Percent	*P*-value	SE	Days	*P*-value	SE
Intercept β_0_	68.3		5.3	11.5		0.4
Trend before intervention β_1_	−1.3	ns	0.9	−0.07	ns	0.1
Effect one month after intervention β_2_	14.1	ns	8.5	−1.4	0.04	0.9
Effect six month after intervention β_2_	45.4	0.002	11.0	0.57	ns	0.8
Trend change after intervention β_3_	4.1	0.02	2.9	0.27	0.03	0.14

Trends pre- and post intervention, and effect of the intervention on prescribing one and six months after the intervention (level change) are reported

^a^Comprise benzylpenicillin in monotherapy, benzylpenicillin in combination with gentamicin, or amoxicillin/ampicillin, ns; data is considered non-significant when *P* > 0.05, *SE* Standard error

In antibiotics categorized as appropriate, 90.9 % and 82.9 % of the prescribing in the pre-intervention audit was maintained during first three days and during entire hospital stay, respectively. For both variables the prevalence of change was even lower post-intervention.

### Total treatment duration

**Fig. 3:**
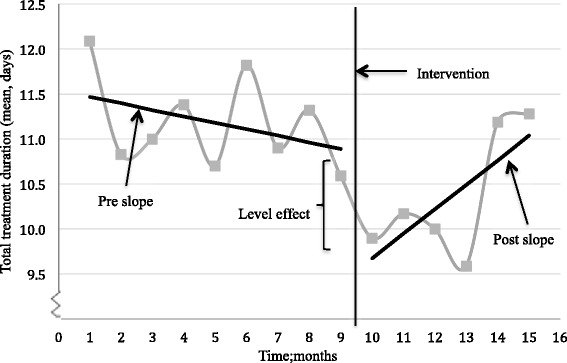
Trend and level change in monthly mean total treatment duration, pre- and post-intervention. Total treatment duration was defined as in-hospital prescribing plus length of prescription at discharge. Pre-intervention audit started January 2014 (point no. 1), the intervention was performed late September 2014 (point no. 9) and the post-intervention audit ended March 2015 (point no. 15)

During the feedback we agreed with the physicians to target a reduction in total treatment duration. Following the intervention, overall mean total treatment duration decreased from 11.2 to 10.4 days, *P* = 0.015. Based on the monthly mean total treatment duration, the ITS showed a non-significant decreasing trend in mean total treatment duration in the pre-intervention phase (−0.07 days per month, *P* > 0.05). Post-intervention, there was an immediate and significant reduction in mean total treatment duration (estimated level change; −1.4 days, *P =* 0.04). However, post-intervention the trend of mean total treatment duration significantly increased (trend change; + 0.27 days per month, *P* = 0.03), and six months post-intervention the effect of the intervention was no longer significant and back to pre-intervention level (estimated level change; +0.57 days, *P > 0.05).* See Fig. 3 and Table 4.

### Dose of benzylpenicillin

The physicians agreed on targeting a change in prescribing from high to low dose of benzylpenicillin. Following the intervention, the proportion of patients prescribed high-dose benzylpenicillin decreased from 48.8 to 38.6 %, *P* = 0.125.

## Discussion

In a Norwegian hospital setting, an A&F intervention combined with distribution of a pocket version of the national CPG led to a substantial and sustained increased prescribing of appropriate empirical antibiotics. A significant immediate reduction on total treatment duration was transitory and vanished six months post-intervention. With the intervention, we achieved a 10 percentage-point targeted reduction in prescribing of high-dose benzylpenicillin.

### Empirical antibiotic prescribing

To halt development and spread of AMR, appropriate prescribing of antibiotics in addition to effective infection control programmes, is important [7]. Increasing levels of extended-spectrum beta-lactamase producing gram negative bacteria has been observed in Norwegian hospitals the recent years, probably associated with import of resistant strains, either in patients or food, and possibly also increasing domestic use of cephalosporines and fluroquinolones [7]. Consequently, testing different intervention approaches to reduce use of known drivers for resistance is important. Particularly, use of cephalosporines and fluroquinoles can result in co-selection of resistance to aminoglycosides, and threaten the Norwegian strategy with extensive use of benzylpenicillin and gentamicin [17]. Cefotaxime is a part of the Norwegian CPG recommendations for severely ill CAP-patients and it can therefore be discussed whether it was correct categorising cephalosporines as inappropriate and tailor a reduction in use. In theory, an intervention to reduce cephalosporines and other inappropriate antibiotics could have resulted in undertreatment. During the feedback session, the ID-specialist highlighted the importance of preferring benzylpenicillin plus gentamicin before cefotaxime, but also emphasised that cefotaxime is an alternative in patients with severe infection and specific complexities. Indicated by CRB-65 score, few patients had a severe infection, and this suggest that cefotaxime might only be necessary in a minority of patients. Altogether, we substantially reduced prescribing of broad-spectrum antibiotics known to promote AMR, and we succeeded in reducing prescribing of these antibiotics with no obvious negative effect on measured clinical outcomes. Consequently, our results indicate that an A&F intervention can have positive effect on empirical antibiotic prescribing and may be expanded to other settings with low level of AMR.

The high degree of maintained empirical prescribing suggests a low level of treatment failure. In other studies, change of empirical prescribing has been reported in 6-31 % of patients [18–20].

The significant higher proportion of patients with penicillin allergy in the pre-intervention phase may theoretically have resulted in a higher prevalence of inappropriate antibiotics prescribing in the pre-intervention phase (i.e. cephalosporins for non-immediate allergy), which again may have biased our positive results post-intervention. To test this, we excluded all patients with penicillin allergy in both phases, which yielded no significant change of the reported mean, trend- or level effect. It is worth noticing that the overall prevalence of penicillin allergy in our study was 12.6 and 5.8 % pre-and post-intervention, which is far higher than the 1 % estimated prevalence of penicillin allergy [21]. In future, patients erroneously labelled penicillin allergic should be identified as it may restrain patients from more appropriate narrow-spectrum antibiotics [21].

The lower proportion of AECOPD in the post-intervention phase compared to the pre-intervention phase did not influence our results as empirical prescribing of both conditions is categorized as appropriate.

### Total treatment duration

Short-term antibiotic treatment has in randomized controlled trials (RCTs) shown the same efficacy as long-term treatment with regards to clinical-, bacteriological- and radiological success [22]. Reducing total treatment duration is important, both to increase patient compliance, reduce risk of adverse effects and to reduce AMR development [23]. For beta-lactams, 3–5 days treatment has been found to be safe [22, 24]. In a European multicentre study including 14 centres from three countries, mean duration was 8.9 and 11 days for AECOPD and CAP patients, respectively [9]. Appropriate treatment duration of antibiotics seems to be neglected and interventions are requested [23, 25].

Multifaceted prospective interventions by antimicrobial stewardship teams have been shown effective in order to reduce length of antibiotic treatment. Avdic et al. showed that a team comprising an ID-specialist and a clinical pharmacist recommended reduction of duration of antibiotic treatment in 59 % of patients by applying an algorithm suggesting the appropriate duration. The mean treatment duration was reduced from 10 to 7 days [26]. Lesprit et al. showed a reduction in mean antibiotic treatment duration from 10 to 7 days in a multicentre RCT in surgical and medical departments, where an ID-specialist performed systematic reviews of antibiotic prescribing at day 1 and day 3–4 in the intervention group. No negative effects on clinical outcomes were observed [27]. In a study by Murray et al., they recommended antibiotic duration based on a severity score, automatic stop dates at time of initiating empirical prescribing and pharmacist feedback to prescribers. Overall, the mean duration of treatment was reduced from 8.3 to 6.8 days, with a subsequent reduction of gastrointestinal adverse effects [28]. A retrospective A&F probably requires fewer recourses compared to prospective A&Fs, and we therefore found it important to test this approach*.* In our study, we achieved a smaller reduction in total antibiotic duration compared with studies based on more comprehensive intervention strategies. In future, prospective interventions involving a multidisciplinary approach should be tested in a Norwegian setting.

### Dose of benzylpenicillin

In Norway, there have been concerns about an unjustified increase in prescribing of high-dose benzylpenicillin over the recent years [29]. High doses are associated with increased overall consumption of antibiotics, which may increase risk of adverse effects and AMR development [30–33]. Benzylpenicillin possesses time dependent killing, and time above the minimum inhibitory concentration (T > MIC) should cover about 50 % of the dosing interval. Peak efficacy is reached at about 5 times above MIC [34]. Based on a national high proportion of susceptible *S.pneumoniae* (MIC ≤ 0.06 mg/L), most patient will benefit 1.2 g x 4 (i.e. low dose) [7]. For *H.influenzae* a clinical breakpoint for benzylpenicillin has not been defined. Time-kill experiments suggested that 3.0 g x 4 (i.e. high dose) can be recommended in *H.influenzae* without resistance mechanisms [35]. However, as *H.influenzae* rarely results in an invasive infection and is expected in a minor proportion of patients, the Norwegian CPG have chosen to recommend low-dose benzylpenicillin as empirical treatment in non-severe patients [6]. It is relevant to notice that in case of identified *H.influenzae,* a change to high-dose benzylpenicillin (when susceptible) or to an alternative antibiotic may be warranted. In severely ill CAP-patients, 3.0 g x 4 is recommended due to possible altered volume of distribution and protein binding. In our study population, only 6 and 11 % (pre- and post-intervention) of the CAP-patients had a severe infection according to CRB-65. Accordingly, we suspect there is a potential for improvement beyond 10-percentage point decline in high-dose benzylpenicillin achieved in this study. However, this must be further explored.

### Behaviour change

At UNN Tromsø, the local infection-, microbiology- and infection control team have focused upon the importance of appropriate empirical antibiotic prescribing over several years. This continuous focus may have lowered the odds of our A&F intervention to increase appropriate empirical antibiotic prescribing. Opposite, there has been little focus on treatment duration and dose, where we observed a lower effect of our intervention. Avdic et al*.* assessed prescribers’ knowledge and practice related to antibiotic prescribing of hospitalized CAP-patients. They observed that most prescribers were uncomfortable with short treatment duration, and suspect that prescribers are not up to date on studies supporting short-time treatment [26]. This is supported in our study by comments received during feedback session. Physicians stated that possible explanations on the discrepancies between audit results and CPG recommendations included that they were not up to date on all CPG recommendations and that prescribing often were based on traditions (i.e. unaware that doxycycline is not recommended for AECOPD and unaware of recommended treatment duration). Qualitative studies on behaviour are important in order to identify barriers and tailor interventions.

### Strengths, limitations and lessons learned

ITS design is the “the strongest quasi-experimental design to evaluate longitudinal effects of time-delimited interventions” [10]. The advantage with ITS, compared to using before-after studies only, is that the trend pre-intervention is accounted for, sustainment of the effect is explored and graphical presentation facilitate interpretation of results [36]. Applying a before-after analysis would have deprived us the opportunity to reveal the non-sustainable effect on treatment duration. In a Cochrane review, it is recommended to include 12 data points both pre-and post-intervention in order to adequately evaluate seasonality, and to assess the immediate effect and sustainability of the intervention [10]. Moreover, increasing number of observations per data point reduces variance and provides more stable estimates. Pre-intervention, the trend is non-significantly decreasing for both empirical antibiotic prescribing and total treatment duration, which we cannot rule out is due to variance (fewer patients admitted during summer months), seasonality or that prescribing is affected by other factors such as deputies at work during summer months. Altogether, there exist some limitation on internal validity in our study. However, the ITS seemed sensitive to detect change in level and trend. Potential bias in form of competing interventions cannot be ruled out without a control to establish our hypothesis. Data collection was not affected by the intervention and outcome variables were objectively assessed.

Another strength in our study is that the physicians at the study department were not informed about the pre-intervention audit before we presented the audit results. Consequently, Hawthorne effects can be ruled out. With regard to the influence on prescribing results, we retrospectively acknowledge that our results could have been even better if we had defined explicit targets together with the department physicians (e.g. “the target is to shortening the mean total treatment length to 7 days”). In addition, providing the feedback both in oral and written format more frequently, as recommended in a recent Cochrane review, could have been beneficial [11]. Electronic prescribing and medication charts are not implemented in the majority of Norwegian hospitals. Consequently, electronic audit and surveillance is currently impossible and limits the possibility for feeding back results as the study progresses. As a consequence of only providing the feedback once and only orally, we could not reach all physicians by our intervention. Despite this, we believe that for instance junior physicians in the emergency department (ED) anyway has been exposed to advices based on our intervention, as they frequently turn to the physicians at the respiratory medicine department when handling CAP and AECOPD patients. These ED-physicians were not invited to the feedback session. In addition, junior staff at the clinical departments rotates every 4–6 months. Consequently, some that are active during the pre- and post-intervention phase may not have been present at the feedback session. However, we emphasize the recommendation from literature that interventions are recommended to target senior physicians, and not junior physicians, as the seniors act as supervisors for the less-experienced physicians [37, 38]. Moreover, lack of adherence to CPGs is more pronounced among seniors compared to juniors [37].

It is relevant to notice that in this study an ID-specialist had a central role in the intervention team. In Scandinavian countries the ID-specialist is perceived as important for antibiotic prescribing [37]. This is in contrast to countries/settings with a more hierarchical work system, where it has been found that clinical leaders and senior physicians overrule the ID-specialists’ advice on antibiotic prescribing [38]. Altogether, it is a limitation that we only provided the feedback once, but a possible negative effect of this is probably reduced by high attendance of senior physicians at the feedback (four out of five attended) and the active participation of the ID-specialist.

We emphasise that CPG recommendations does not address all patient scenarios, and complete adherence to CPG is probably not desirable. In this study, examples of justified non-adherence are use of cefotaxime in patients with severe infection and specific complexities, empirical antibiotic prescribing in patients with penicillin allergy, microbiological findings and delayed clinical response. We have not collected data on this, but it should be considered for future studies.

Our study has other limitations. First, our study has limited external validity. Optimally the intervention should have been performed in multiple hospitals. Second, economic and microbiological measures where not assessed in this study, and should if possible, be included in future studies. Third, proportion of intensive care units admissions pre- and post-intervention is also an alternative outcome measure that should be collected to describe severity of disease in the patient population. Fourth, CRB-65, which is a well-validated severity-scoring tool, was not documented in patient records and we therefore calculated the severity based on information at admission. Fifth, we are unable to distinguish the effect of our A&F intervention from the effect of the pocket version of the national CPG that was distributed to all departments at the hospital during same month as our intervention. Preceding the distribution of this pocket version, the national CPG was published online 2013 [6]. One the other hand, as literature indicate that distribution of educational material alone is ineffective, we do not believe that the effect of this pocket version may have been substantial in itself [39].

## Conclusion

A&F is an essential part of antibiotic stewardship programs, and testing this design in a setting with low AMR is important. We have demonstrated improved and sustained prescribing of appropriate empirical antibiotics by combining an A&F intervention with distribution of a pocket version of CPG recommendations. The intervention did not have any obvious negative effects on clinical outcomes. The intervention led to reduced prescribing of inappropriate antibiotics, such as third generation cephalosporins and doxycycline. Our results indicate that A&F may be suitable for reducing prescribing of broad-spectrum antibiotics, and the design can potentially be expanded to other low AMR-settings. Following the intervention, we observed a significant immediate reduction in total treatment duration, however this effect was not sustained after six months. The 10-percentage point reduction in prescribing of high dose benzylpenicillin was not significant, but we suspect there is a potential for improvement beyond 10-percentage point. In order to sustain appropriate total treatment duration, defining explicit targets, and providing the feedback both in oral and written format more frequently should be tested in future A&F-studies. In addition, more comprehensive interventions should be tested. Prospective interventions with antibiotic stewardship teams comprising an ID-specialist and a clinical pharmacist have shown to be successful in these aspects.

## Additional files

Additional file 1:
**Differences in study outcome measures for appropriate and inappropriate empirical antibiotics (** ***n*** **= 408).** (PDF 81 kb) Additional file 2:
**Bacterial findings and contribution of different methods to diagnostic yield.** (PDF 82 kb) Additional file 3:
**Antibiotic sensitivity patterns for culture isolates.** Antibiotic sensitivity patterns for *S.pneumoniae* and *H.influenzae* in inpatients with Community-Acquired Pneumonia or Acute Exacerbation of Chronic Obstructive Pulmonary Disease. (PDF 78 kb) Additional file 4:
**Detailed information on treatment versus outcome for patients with identified pathogens (** ***n*** **= 92).** (PDF 131 kb)

## Abbreviations

A&F: Audit and feedback
AECOPD: Acute exacerbation of chronic obstructive pulmonary disease
AMR: Antimicrobial resistance
CAP: Community-acquired pneumonia
CPG: Clinical practice guideline
CRB-65: Confusion, respiration, blood pressure and age ≥ 65y
ED: Emergency department
ID-specialist: Infection disease-specialist
ITS: Interrupted time series
MIC: Minimum inhibitory concentration
RCT: Randomized controlled trial
UNN: University hospital of North Norway
