# Supplementary material for: An audit and feedback intervention study increased adherence to antibiotic prescribing guidelines at a Norwegian hospital
Source: BMC Infect Dis. 2016 Feb 27;16:96. doi: 10.1186/s12879-016-1426-1 (PMC4769530; doi:10.1186/s12879-016-1426-1)
Supplement: Additional file 1: — Differences in study outcome measures for appropriate and inappropriate empirical antibiotics ( n = 408). (PDF 81 kb) [file 12879_2016_1426_MOESM1_ESM.pdf]

Additional file 1: Differences in study outcome measures for appropriate and inappropriate empirical antibiotics (n=408)

	Appropriate empirical antibiotic treatment	Inappropriate empirical antibiotic treatment	*P*-value
Length of stay in hospital	5.6 days	5.5 days	NS
30-day readmission	50/268 (18.7%)	29/117 (24.8%)	NS
30-day mortality	24/286 (8.4%)	8/122 (6.6%)	NS
CRB-65			NS
0	31/190 (16.3%)	10/69 (14.5%)	
1	73/190 (38.4%)	26/69 (37.7%)	
2	58/190 (30.5%)	22/69(31.9%)	
3	11/190 (5.8%)	7/69 (10.1%)	
4	2/190 (1.1%)	1/69 (1.4%)	
MD	15/190 (7.9%)	3/69 (4.3%)	
Total treatment duration	10.9 days	10.8 days	NS

Note: Study outcome measures (and CRB-65) for appropriate (i.e. benzylpenicillin in monotherapy or in combination with gentamicin, or ampicillin/amoxicillin) and inappropriate (i.e. all other antibiotics) empirical antibiotic treatment is tested on all 408 patients. Subgroup-analysis on patients with identified pathogens (n=92), also provide non-significant results.

CRB-65; Confusion, respiration, blood-pressure and age ≥ 65 years (only calculated for patients with community-acquired pneumonia), MD; missing data, NS; data is considered non-significant when P>0.05, tested with Chi-square test of Student´s t-test.
