# Supplementary material for: An audit and feedback intervention study increased adherence to antibiotic prescribing guidelines at a Norwegian hospital
Source: BMC Infect Dis. 2016 Feb 27;16:96. doi: 10.1186/s12879-016-1426-1 (PMC4769530; doi:10.1186/s12879-016-1426-1)
Supplement: Additional file 2: — Bacterial findings and contribution of different methods to diagnostic yield. (PDF 82 kb) [file 12879_2016_1426_MOESM2_ESM.pdf]

Additional file 2: Bacterial findings and contribution of different methods to diagnostic yield

Pathogen	No. of patients with positive findings	Blood culture	Urine antigen test	BAL/ pleural fluid	Sputum culture	Throat culture	NP culture	PCR from NP culture	Serology
Streptococcus pneumoniae	46	2	35	NA	13	5	5	NA	NA
Haemophilius influenzae	25	NA	NA	3	15	3	4	NA	NA
Pseudomonas aeruginosa[a]	11	1	NA	NA	9	2	1	NA	NA
Staphylococcus aureus	5	2	NA	NA	3	1	1	NA	NA
Klebsiella pneumoniae	3	NA	NA	NA	4	NA	NA	NA	NA
Moraxella catarrhalis	4	NA	NA	NA	3	1	NA	NA	NA
Mycoplasma pneumoniae	2	NA	NA	NA	NA	NA	NA	1	1
Streptococcus pyogenes (GAS)	1	NA	NA	NA	NA	1	NA	NA	NA
Total[b]	97	6	33	3	49	12	11	1	1

Note: Some patients had aetiology established by use of different methods.
BAL; bronchoalveolar lavage; NA; not applicable/not tested, NP; nasopharynx, PCR; polymerase chain reaction
[a] *Pseudomonas aeruginosa* was identified in 7 different patients (two patients had multiple admissions where the specific pathogen was identified)
[b] Five patients had multiple pathogens; a total of 97 pathogens were identified in 92 patients.
