# Supplementary material for: An audit and feedback intervention study increased adherence to antibiotic prescribing guidelines at a Norwegian hospital
Source: BMC Infect Dis. 2016 Feb 27;16:96. doi: 10.1186/s12879-016-1426-1 (PMC4769530; doi:10.1186/s12879-016-1426-1)
Supplement: Additional file 3: — Antibiotic sensitivity patterns for culture isolates. Antibiotic sensitivity patterns for S.pneumoniae and H.influenzae in inpatients with Community-Acquired Pneumonia or Acute Exacerbation of Chronic Obstructive Pulmonary Disease. (PDF 78 kb) [file 12879_2016_1426_MOESM3_ESM.pdf]

Additional file 3: Antibiotic sensitivity patterns for culture isolates.
Antibiotic sensitivity patterns for *S.pneumoniae* and *H.influenzae* in inpatients with Community-Acquired Pneumonia or Acute Exacerbation of Chronic Obstructive Pulmonary Disease.

**Antibiotic**	***S.pneumoniae* (n=22)**			***H.influenzae* (n=25)**		
	**S**	**I**	**R**	**S**	**I**	**R**
Penicillin	14/22	8/22	-	-	-	-
Ampicillin	14/15	1/15	-	20/25	-	5/25
Erythromycin	17/22	-	5/22	-	-	-
Cefuroxime	14/14	-	-	23/24	-	1/24
Cefotaxime	19/22	3/22	-	13/24	-	11/24

S; Sensitive, I; Intermediate sensitive, R; resistant
