# Supplementary material for: An audit and feedback intervention study increased adherence to antibiotic prescribing guidelines at a Norwegian hospital
Source: BMC Infect Dis. 2016 Feb 27;16:96. doi: 10.1186/s12879-016-1426-1 (PMC4769530; doi:10.1186/s12879-016-1426-1)
Supplement: Additional file 4: — Detailed information on treatment versus outcome for patients with identified pathogens ( n = 92). (PDF 131 kb) [file 12879_2016_1426_MOESM4_ESM.pdf]

Additional file 4: Detailed information on treatment versus outcome for patients with identified pathogens (n=92).

ID-no	Empirical antibiotic	CRB-65 score	Pathogen	Total treatment duration	Length of stay	30-day mortality (0;no, 1;yes)	30-day readmission (0;no, 1;yes)
1	Cefuroxime	MD	*S.pneumoniae*	13	6	0	0
2	Benzylpenicillin	2	*S.pneumoniae*	13	3	0	1
3	Benzylpenicillin + gentamicin	1	*S.pneumoniae*<br>*M.catarrhalis*	9	3	0	0
4	Benzylpenicillin + gentamicin	1	*S.pneumoniae*	14	10	0	0
5	Benzylpenicillin	NA	*S.pneumoniae*<br>*P.auerginosa*	9	3	0	1
6	Benzylpenicillin	0	*S.pneumoniae*	10	6	0	0
7	Benzylpenicillin	0	*S.pneumoniae*	11	1	0	1
8	Benzylpenicillin	NA	*S.pneumoniae*	10	3	0	0
9	Doxycycline	NA	*S.pneumoniae*	12	17	0	0
10	Benzylpenicillin	3	*S.pneumoniae*	-	-	1	-
11	Doxycycline	NA	*S.pneumoniae*	9	1	0	1
12	Benzylpenicillin	1	*S.pneumoniae*	14	6	0	1
13	Benzylpenicillin	1	*S.pneumoniae*	15	9	0	0
14	Benzylpenicillin	2	*S.pneumoniae*	14	4	0	0
15	Cefotaxime	3	*S.pneumoniae*	30	21	0	0
16	Benzylpenicillin	1	*S.pneumoniae*	11	4	0	0
17	Benzylpenicillin + gentamicin	1	*S.pneumoniae*	14	7	0	0
18	Cefotaxime	3	*S.pneumoniae*	13	9	0	0
19	Benzylpenicillin + gentamicin	2	*S.pneumoniae*<br>*M.catarrhalis*	10	6	0	1
20	Benzylpenicillin	1	*S.pneumoniae*	14	3	0	0
21	Benzylpenicillin	1	*S.pneumoniae*	11	-	1	-
22	Doxycycline	MD	*S.pneumoniae*	11	8	0	0
23	Benzylpenicillin	1	*S.pneumoniae*	-	.	1	-
24	Benzylpenicillin	NA	*S.pneumoniae*	15	8	0	0

25	Benzylpenicillin	2	*S.pneumoniae*	18	34	0	1
26	Benzylpenicillin	MD	*S.pneumoniae*	9	15	0	0
			M.catarrhalis				
27	Benzylpenicillin	1	*S.pneumoniae*	9	3	0	0
28	Ceftriaxone	2	*S.pneumoniae*	14	19	0	0
29	Amoxicillin	NA	*S.pneumoniae*	7	3	0	1
30	Benzylpenicillin	3	*S.pneumoniae*	11	12	0	0
31	Benzylpenicillin + gentamicin	1	*S.pneumoniae*	12	3	0	1
32	Benzylpenicillin + gentamicin	1	*S.pneumoniae*	11	4	0	1
33	Benzylpenicillin	1	*S.pneumoniae*	12	8	0	0
34	Benzylpenicillin	2	*S.pneumoniae*	15	8	0	0
35	Benzylpenicillin + gentamicin	1	*S.pneumoniae*	-	-	1	-
36	Benzylpenicillin	2	*S.pneumoniae*	9	2	0	0
37	Amoxicillin	NA	*S.pneumoniae*	5	4	0	0
38	Benzylpenicillin	3	*S.pneumoniae*	19	10	0	0
39	Benzylpenicillin	NA	*S.pneumoniae*	9	4	0	0
40	Benzylpenicillin	MD	*S.pneumoniae*	9	4	0	0
41	Benzylpenicillin + gentamicin	3	*S.pneumoniae*	-	-	1	-
42	Benzylpenicillin	3	*S.pneumoniae*	11	5	0	0
43	Benzylpenicillin + ciprofloxacin	2	*S.pneumoniae*	-	-	1	-
			S.aureus				
44	Meropenem	3	*S.pneumoniae*	11	8	0	1
45	Cefotaxime	4	*S.pneumoniae*	9	-	1	-
46	Benzylpenicillin	NA	*S.pneumoniae*	8	4	0	0
47	Benzylpenicillin + gentamicin	1	*H.influenzae*	16	9	0	0
48	Benzylpenicillin	2	*H.influenzae*	15	6	0	0
49	Cefotaxime	1	*H.influenzae*	11	4	0	0
50	Erythromycin	1	*H.influenzae*	9	6	0	1
51	Benzylpenicillin	1	*H.influenzae*	15	8	0	0
52	Benzylpenicillin	1	*H.influenzae*	13	3	0	0
53	Doxycycline	1	*H.influenzae*	12	4	0	1
54	Benzylpenicillin	2	*H.influenzae*	14	4	0	0

55	Benzylpenicillin	0	*H.influenzae*	10	3	0	0
56	Doxycycline	NA	*H.influenzae*	10	4	0	0
57	Doxycycline	NA	*H.influenzae*	9	2	0	1
58	Cefotaxime	1	*H.influenzae*	11	4	0	0
59	Amoxicillin	NA	*H.influenzae*	7	3	0	0
60	Benzylpenicillin	2	*H.influenzae*	15	9	0	0
61	Gentamicin + clindamycin	NA	*H.influenzae*	10	5	0	0
62	Benzylpenicillin	MD	*H.influenzae*	10	5	0	0
63	Amoxicillin	NA	*H.influenzae*	10	9	0	0
64	Benzylpenicillin + gentamicin	2	*H.influenzae*	10	9	0	0
65	Benzylpenicillin	MD	*H.influenzae*	-	-	1	-
66	Benzylpenicillin	2	*H.influenzae*	12	1	0	0
67	Benzylpenicillin	3	*H.influenzae*	14	34	0	0
68	Benzylpenicillin	1	*H.influenzae*	10	3	0	1
69	Benzylpenicillin	1	*H.influenzae*	15	8	0	0
70	Benzylpenicillin	2	*H.influenzae*	11	4	0	0
71	Ampicillin	3	*H.influenzae*	7	14	0	0
72	Benzylpenicillin	0	*M.pneumoniae*	12	2	0	0
73	Benzylpenicillin	0	*M.pneumoniae*	25	11	0	0
74	Meropenem	1	*K.pneumoniae*	7	7	0	0
75	Meropenem	1	*K.pneumoniae*	5	5	0	1
76	Benzylpenicillin	1	*K.pneumoniae*	12	12	0	0
77	Benzylpenicillin	1	*S.aureus*	13	4	0	0
78	Benzylpenicillin + gentamicin	1	*S.aureus*	14	6	0	1
79	Benzylpenicillin + gentamicin	2	*S.aureus*	13	14	0	0
80	Piperacillin + Tazobactam	1	*P. aeruginosa*<br>*S.aureus*	13	6	0	0
81	Doxycycline	NA	*M. catarrhalis*	16	6	0	1
82	Benzylpenicillin	1	*M. catarrhalis*	15	11	0	0
83	Benzylpenicillin	2	*S.pyogenes*	12	6	0	0
84	Benzylpenicillin	NA	*P. aeruginosa*	15	11	0	0

85	Doxycycline	NA	*P. aeruginosa*	14	14	0	0
86	Benzylpenicillin	2	*P. aeruginosa*	15	4	0	0
87	Amoxicillin	NA	*P. aeruginosa*	15	5	0	1
88	Cefotaxime	1	*P. aeruginosa*	13	3	0	0
89	Cefuroxime	1	*P. aeruginosa*	14	14	0	0
90	Cefotaxime	4	*P. aeruginosa*	-	-	1	-
91	Meropenem	MD	*P. aeruginosa*	12	14	0	0
92	Benzylpenicillin + gentamicin	2	*P. aeruginosa*	12	3	0	1

CRB-65; Confusion, respiration, blood-pressure and age ≥ 65 years, MD; missing data, NA; not applicable for patients with acute exacerbation of chronic obstructive pulmonary disease
